# Regulation of cytokines by small RNAs during skin inflammation

**DOI:** 10.1186/1423-0127-17-53

**Published:** 2010-07-01

**Authors:** Rasmus O Bak, Jacob G Mikkelsen

**Affiliations:** 1 Department of Human Genetics, University of Aarhus, DK-8000 Aarhus C, Denmark

## Abstract

Intercellular signaling by cytokines is a vital feature of the innate immune system. In skin, an inflammatory response is mediated by cytokines and an entwined network of cellular communication between T-cells and epidermal keratinocytes. Dysregulated cytokine production, orchestrated by activated T-cells homing to the skin, is believed to be the main cause of psoriasis, a common inflammatory skin disorder. Cytokines are heavily regulated at the transcriptional level, but emerging evidence suggests that regulatory mechanisms that operate after transcription play a key role in balancing the production of cytokines. Herein, we review the nature of cytokine signaling in psoriasis with particular emphasis on regulation by mRNA destabilizing elements and the potential targeting of cytokine-encoding mRNAs by miRNAs. The proposed linkage between mRNA decay mediated by AU-rich elements and miRNA association is described and discussed as a possible general feature of cytokine regulation in skin. Moreover, we describe the latest attempts to therapeutically target cytokines at the RNA level in psoriasis by exploiting the cellular RNA interference machinery. The applicability of cytokine-encoding mRNAs as future clinical drug targets is evaluated, and advances and obstacles related to topical administration of RNA-based drugs targeting the cytokine circuit in psoriasis are described.

## Introduction

Cytokines are intercellular signaling proteins that serve as key modulators of the immune system and inflammation. Cells respond to extracellular stress or stimuli by operating intracellular signaling cascades that coordinate cellular gene expression through complex networks of kinase activation, protein phosphorylations, and activation of DNA-binding proteins that translate signals at the cell surface to actions of transcriptional regulation of cellular genes. Cytokines modulate the communication between cells of the immune system and between immune cells and differentiated somatic cells. Upon binding to their cognate receptor on the cell surface, cytokines trigger transcriptional changes and balance cellular activities ranging from growth to differentiation and cell survival. Cytokine-directed transcriptional induction of yet other cytokines may further enhance the innate immune response in an increasingly entangled network of signals.

Genome-wide association studies have shown that mutations of genes encoding cytokines, cytokine receptors, or downstream players in the cellular signaling cascades associated with autoimmune disease. Effectors of the different signaling cascades and the transcriptional regulation operated through these pathways have been reviewed at numerous occasions. In this review, we concentrate exclusively on the posttranscriptional mechanisms that act together to balance the expression of cytokines during inflammation. The discovery of RNA interference and the regulatory actions of small RNAs have unveiled a new world of posttranscriptional regulation and yet new layers of complexity in cellular signaling pathways that are in play during inflammation. Small non-coding RNA species, produced from intronic and intergenic regions across the mammalian genome, are key players in post-transcriptional regulatory pathways of gene expression. MicroRNAs (miRNAs) interact with mRNAs and trigger translational suppression or mRNA degradation through recruitment of cellular proteins. Short-lived RNA transcripts, such as several cytokine-encoding mRNAs, contain RNA destabilizing elements and regulatory miRNA binding motifs that may in concert contribute to stringent regulation of cytokine production. Dysregulated cytokine production is a hallmark of tissues affected by chronic inflammatory disease, and miRNAs are likely to play important, but hitherto vaguely characterized, roles in aberrant cytokine regulation and disease development and progression. With emphasis on skin inflammation and psoriasis vulgaris in particular (referred to as psoriasis in the remainder of the review), we focus here on the regulation of cytokines at the RNA level in relation to development of inflammatory disease. We provide an overview of the network of cytokine signaling in psoriasis and its regulation through induced RNA destabilization and miRNA association and the potential linkage between mRNA decay and targeting by miRNA. The applicability of cytokine-encoding mRNAs as future therapeutic targets is evaluated, and obstacles and advances related to topical administration of RNA-based drugs targeting the cytokine circuit in psoriasis are described.

Psoriasis is an idiopathic chronic skin disorder which has been estimated to affect about 2% of the population world-wide [1-3]. The disease manifests in different clinical variants, the most predominant form being plaque psoriasis affecting about 80% of all psoriasis patients. Plaque psoriasis shows as erythematous and scaly lesions that are red or salmon pink in color and often covered by white or silvery scaly plaques [4]. Adding to the physical distress, severe psychosocial aspects of psoriasis can strongly impact the quality of life [5,6]. Histologically, psoriasis displays a thickened epidermis (epidermal hyperplasia), dilated and prominent blood vessels in the dermis caused to some extent by an up-regulation of vascular endothelial growth factor (VEGF), and an inflammatory infiltrate of leukocytes predominantly in the dermis.

The epidermal hyperplasia is associated with underexpression of keratinocyte differentiation markers, which causes incomplete differentiation of keratinocytes (parakeratosis). Histopathologically this shows as retention of nuclei of cells in the stratum corneum (the outer stratified cell layer) and an overall thickening of the epidermis (acanthosis). The transit time of keratinocytes from the basal cell layer of the epidermis to the stratum corneum is reduced from 28 days to about 4-7 days in psoriatic lesions.

Many advances have been made in recent years in unraveling the molecular mechanisms of psoriasis, but many questions still remain unanswered. It is still not known if the primary nature of the condition is epithelial or immunologic and the autoimmune cause of the inflammation is still to be uncovered [7]. Moreover, additional information regarding the relevance of cutaneous versus systemic factors and genetic versus environmental influences on disease initiation is still lacking. However, it is widely accepted that a dysregulation of the immune system by an alteration in the cytokine production provides persistent proinflammatory signals in the skin that lead to psoriatic lesions. This is also supported by the fact that psoriasis may be cured by allogenic bone marrow transplantation [8] and that psoriasis may be transferred from the bone marrow transplant donor to the recipient [9]. Although a model involving dysregulated cytokines is conceptually useful and therapeutically relevant, it does not entail the full complexity of the disease or the more than 1,300 genes that are differentially expressed in psoriatic lesions [10].

### Cytokine networks in psoriasis

**Figure 1:**
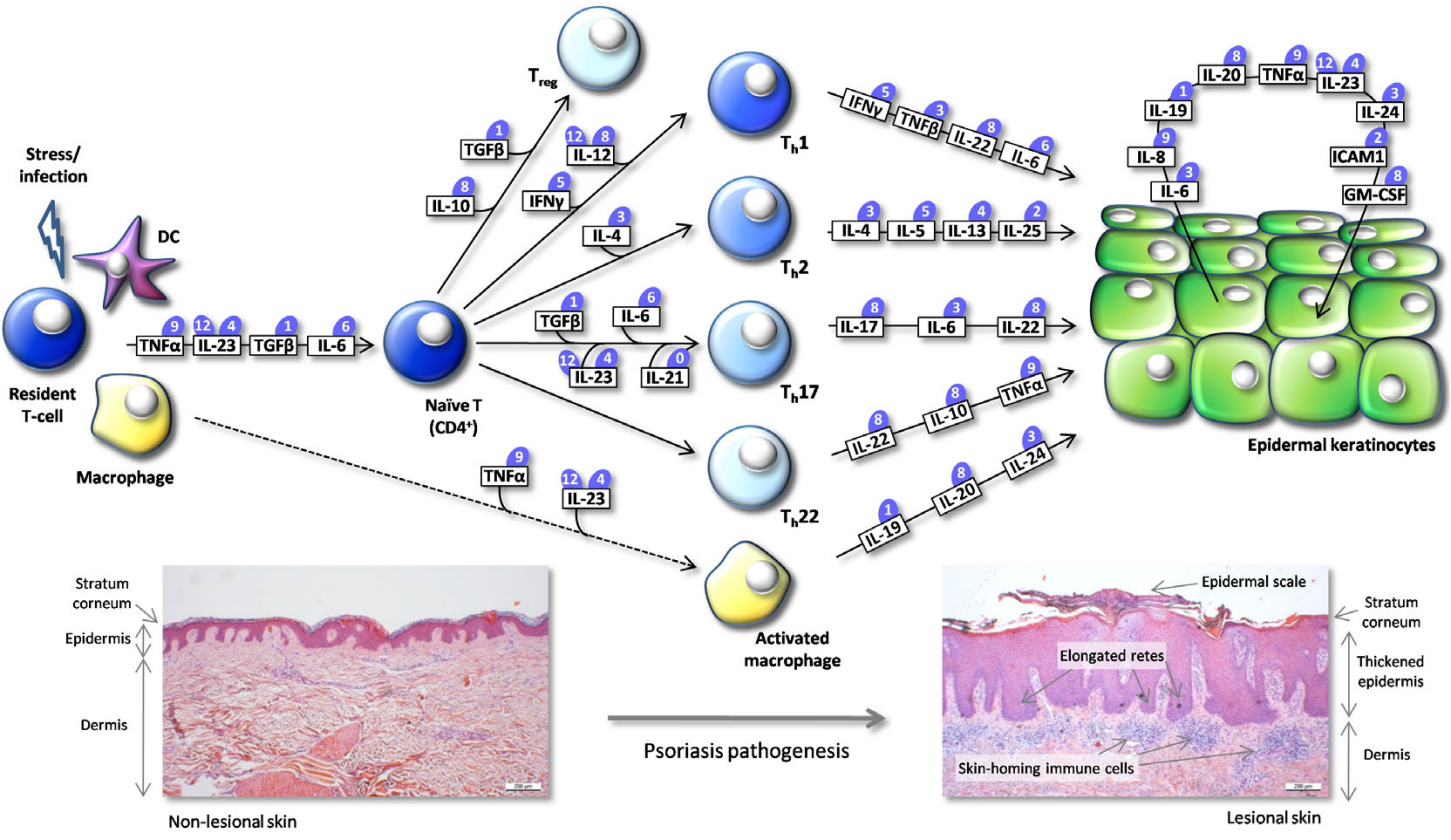
**Schematic representation of the cytokine network driving development of psoriasis**. Cytokines mediate intercellular communication between skin-infiltrating activated immune cells and epidermal keratinocytes. Resident T-cells and antigen-presenting cells are activated upon stimulation by stress or infection (indicated by lightning symbol), leading to production of cytokines, as indicated by horizontal arrow labeled with boxed cytokines. Naïve CD4^+ ^T-cells undergo differentiation upon exposure to different cytokine milieus, as indicated by the listed cytokines driving the differentiation into one of five T-helper (T_h_) cell lineages, T_h_1, T_h_2, T_h_17, T_h_22, or T_reg_. A subset of cytokines (listing is not intended to be absolute), produced by each of the T_h _lineages and macrophages, orchestrates a multi-faceted stimulation of epidermal keratinocytes. This leads to production of an additional set of cytokines triggering epidermal remodeling through altered keratinocyte growth and differentiation as well as angiogenesis. Numbers in blue ovals above cytokine names indicate the number of AUUUA pentamer sequences (potential ARE sequences) that are located in the 3'UTR of each cytokine mRNA (or for heterodimeric cytokines the number of AUUUA sequences in mRNA species encoding each of the two subunits). Also shown is the development of pre-psoriatic, non-lesional skin (left insert) to a psoriatic plaque (right insert) driven by activated immune cells and cytokine-activated keratinocytes. The lesional skin section shows thickened epidermis with elongated retes and an abnormal outer skin layer (stratum corneum) with formation of epidermal scales. Skin-infiltrating immune cells (including activated T_h _cells, dendritic cells, and macrophages) are visible in the dermis.

Psoriasis is the result of an intensifying cross-talk between skin-infiltrating immunocytes and epidermal keratinocytes. An overview of the cell types and primary cytokines involved in this intercellular communication is provided in Figure 1. The leukocyte infiltrate in psoriatic lesions consists primarily of dendritic cells, macrophages, cytotoxic T cells and activated T-helper cells (T_h _cells). Naïve CD4+ T-cells that are activated through interaction with antigen-presenting cells (APCs) - primarily dendritic cells and macrophages - play a dominant role in both initiation and persistence of the disease. Depending on the exposure to different cytokines, naïve T-cells may develop into one of the at least five different CD4+ T-cell lineages that have been identified to date. T_h_1 cells play a key role in clearance of intracellular pathogens, whereas T_h_2 cells trigger and organize a defense response against extracellular pathogens. The T_h_17 and T_h_22 lineages, the latter which was just recently discovered [11], assist the T_h_1 and T_h_2 lineages in inducing an inflammatory response, whereas T_reg _cells serve to limit the immune response by production of immunosuppressive cytokines [12].

According to the classical T_h_1/T_h_2 model by which naïve T_h _cells differentiate into either T_h_1 or T_h_2 cells producing the defining cytokines interferon gamma (IFNγ) and interleukin (IL)-4, respectively, psoriasis was classified as a T_h_1 disease with a marked type 1 cytokine profile [13]. This model was supported by the fact that a shift in the cytokine production from a T_h_1 to T_h_2 cytokine profile was found to ameliorate the disease [14]. However, with the discovery of a new subset of T_h _cells, T_h_17 cells [15], which seem to play a prominent role in psoriasis [13], a new paradigm has taking over with psoriasis being classified as a T_h_1/T_h_17 disease. T_h_17 cells are named according to their production of IL-17, which in turn induces the production of other proinflammatory cytokines, such as TNFα, IL-6, IL-8 and IL-22 (Figure 1) [16]. Identification of the T_h_22 lineage, which is characterized by secretion of IL-22 and TNFα, but does not express IFNγ, IL-4, or IL-17 [11], has caused yet another paradigm shift [17]. T_h_22 cells represent a stable and independent T_h _cell lineage with a transcriptome profile that differs from those of T_h_1, T_h_2, and T_h_17 cells in inflamed skin. Cells of the T_h_22 lineage appear to be induced by Langerhans cells and dermal dendritic cells [18], home preferentially to the epidermis, and are thought to amplify proinflammatory signals induced by TNFα in keratinocytes. Interestingly, there seems to be some plasticity within the population of psoriatic skin-homing T_h _cells. Hence, in addition to distinct populations of T-cells producing IFNγ (T_h_1), IL-4 (T_h_2), IL17 (T_h_17), and IL-22 (T_h_22), respectively, subsets of T_h _cells expressing two or even three of these cytokines can be identified [11]. It has been suggested, therefore, that subsets of CD4+ T_h _cells may not necessarily represent distinct lineages but possibly T_h _cells at different levels of differentiation [19], adding perhaps yet another layer of complexity to the T_h_-directed orchestration of the innate immune response in psoriatic skin.

Sabat and co-workers proposed a model for the pathogenesis of psoriasis consisting of three phases [20]. The sensitization phase includes antigen processing and presentation by primarily dendritic cells and macrophages followed by generation and mobilization of activated T-cells. A silent intervening phase is followed by the effector phase in which CD4+ and CD8+ T-cells and other immune cells migrate to the skin and here undergo further activation and proliferation. Keratinocytes are involved in the cytokine-mediated inflammation by responding to and producing cytokines. Cytokine-stimulated keratinocytes are activated, and this process leads to epidermal remodeling with increased proliferation and dysregulated differentiation of keratinocytes. The keratinocyte-produced cytokines (a subset is listed in Figure 1) have several functions including amplification of immune cell trafficking, immune cell adhesion to endothelial cells and promotion of angiogenesis [21]. Additionally, keratinocytes produce antimicrobial peptides which have chemotactic as well as immune cell modulating functions [22]. These are just some of the functions of keratinocytes in psoriasis pathogenesis which further complicate the issue regarding the psoriatic dogma of epidermal keratinocytes versus immune cells as being the main contributor to disease initiation and continuance.

### Current therapeutic strategies targeting the cytokine network in psoriasis

Current therapies for psoriasis include topical agents, phototherapy and systemic therapies. Topical therapies are mainly used for mild psoriasis and in addition to the widely used corticosteroids, keratolytic ointments, topical retinoids and vitamin D analogues are also used [23]. Phototherapy and systemic therapies are mostly used in moderate to severe psoriasis. The systemic therapeutic approach is mainly based on modulating either keratinocyte proliferation, T-cell activities or key players in the cytokine network [24]. Common non-biological systemic therapies include ciclosporin, methotrexate, oral retinoids and psoralen. Biological therapeutics, in contrast, are relatively new in psoriasis treatment and are currently based on recombinant receptors or antibodies targeting either T-cell function or cytokines [25].

In an extremely complex network of cytokine-directed intercellular signaling, different subsets of cytokines seem crucial at different stages of the pathogenesis. TNFα play a key role during naïve T-cell activation, whereas IFNγ, IL-12, IL-4, IL-6, TGF-β, and IL-23 are prime mediators of T_h _differentiation (Figure 1). T_h_-derived IFNγ, IL-6, IL-17, IL-22, and TNFα as well as several cytokines derived from dendritic cells and macrophages are prominent regulators of the keratinocyte response which will in turn result in the production of a number of cytokines including IL-6, IL-8, IL-20, and IL-23 (Figure 1). All cytokines in the pathogenesis may in theory represent useful therapeutic targets. Inhibitors of TNFα as an absolute key player have been applied to psoriasis in animal models and/or patients with great success. Especially TNFα inhibitors such as Infliximab, Etanercept, and Adalimunab have proven their worth in inflammatory conditions such as rheumatoid arthritis, Crohn's disease and ulcerative colitis [26]. More recently, systemic administration of such inhibitors of TNFα has been established as a potent treatment of severe psoriasis [27,28].

More recently, drugs simultaneously targeting IL-12 and IL-23 have been developed. Both IL-12 and IL-23 are up-regulated in psoriatic lesional skin [29]. As heterodimeric cytokines, they share the subunit p40 encoded by the IL-12B gene. Both interleukins are expressed by dendritic cells and macrophages, but also to some extent by keratinocytes [30,31]. IL-12 is produced early after APC activation and works in a paracrine fashion by stimulating natural killer cells to produce IFNγ and on naïve T-cells to drive their differentiation into T_h_1 cells [32]. Thereby, IL-12 is considered an important factor in initiating and driving the T_h_1 cytokine profile that is present in psoriasis. IL-12 can also work in an autocrine fashion in which APC function is enhanced and contributes to IFNγ production [33]. IL-23, in contrast, does not appear to have a direct effect on naïve T-cells. However, this cytokine seems to have a crucial role in the survival and proliferation of T_h_17 cells, thereby promoting IL-17 production. In fact, a study shows that IL-17 and IFNγ display a marked synergism in the stimulation of IL-6 and IL-8 production by human keratinocytes [34]. This suggests that IL-12 and IL-23, via their function in T_h_1 and T_h_17 cell development, synergistically amplify inflammation through stimulation of keratinocytes to augment their secretion of proinflammatory cytokines. Interestingly, genetic polymorphisms in IL-12B and one of the IL-23 receptor subunits (IL-23R), have been linked to psoriasis [35] and many of the current therapies used in treating psoriasis, such as narrow-band UVB therapy [36], Etanercept (soluble TNFα receptor) [37] and Alefacept (an antagonist of T cell activation) [38] all reduce levels of IL-23. The shared p40 subunit of IL-12 and IL-23 was confirmed as a potent therapeutic target with the approval of Ustekinumab, a subcutaneously injected monoclonal antibody directed against p40. In fact, in a phase III trial, Ustekinumab showed to be more effective and to require fewer injections than the TNFα-inhibitor Etanercept [39].

In spite of the recent success of biological cytokine-targeting drugs, there is still a continued need for psoriasis treatments that are safe, effective and convenient. Patients may respond differently to biological agents, and relapses may occur in some patients. Furthermore, there is an increased occurrence of opportunistic infections associated with the general immunosuppressive action of these highly potent cytokine inhibitors. Hence, more evidence is needed concerning the long-term safety of such biological agents. However, the encouraging clinical experience with anti-cytokine treatment has prompted the search for and development of inhibitors of other psoriasis-related cytokines. As a recent example of this, findings in a xenotransplantation mouse model suggest that blockage of IL-20 signaling by systemic administration of anti-IL20 antibodies leads to resolution of psoriasis [40].

### Regulation of the cytokine network at the RNA level

The regulation of cytokine production is the very foundation of the innate immune response during stress, infection, and injury. Simultaneous transcriptional regulation by several sets of genes affected by cellular signaling pathways is conceived by a network of transcription factors, co-regulators, and epigenetic moderators. Signaling routes and transcriptional control have been covered in numerous reviews (see for example enlightening reviews in references [41-43], and it is beyond the scope of this review to cover such regulation. Instead, we will focus here on cutaneous posttranscriptional regulation of cytokine genes and the potential targeting of cytokine-encoding mRNAs as a new therapeutic approach.

#### Decay of cytokine-encoding mRNAs triggered by AU-rich element

**Figure 2:**
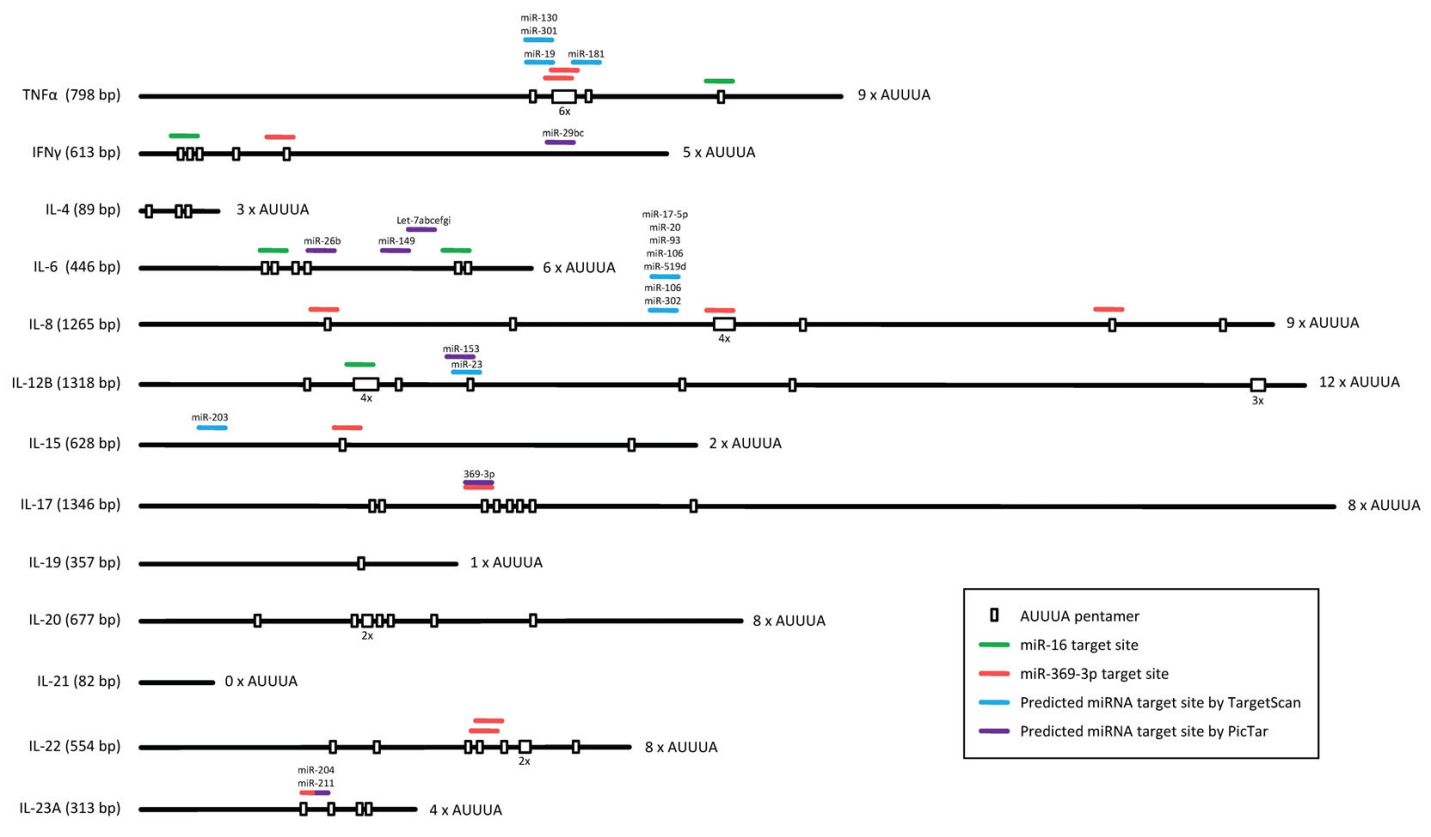
**Overview of pentameric ARE motifs and potential miRNA target sequences in psoriasis-relevant cytokines**. The pentameric sequence motif, AUUUA, has been shown to be a discriminating motif for AREs which generally consist of clustered and/or overlapping pentamer repeats. miR-16 and miR-369-3p target sequence motifs in the ARE of TNFα, which are also present in multiple psoriasis-relevant cytokines as indicated by green and red bars, respectively. Target sites are shown by blue and purple bars, respectively, for miRNAs that are predicted by the PicTar (pictar.mdc-berlin.de) and TargetScan (targetscan.org) algorithms to target psoriasis-relevant cytokines.

Many cytokine mRNAs are expressed as short-lived transcripts. This instability is often conferred by functional AU-rich elements (AREs) which are adenosine- and uridine-rich sequences in the 3' UTR of not only multiple cytokines, but also of numerous other genes - as much as 5-8% of human genes [44]. Computational predictions have suggested that approximately half of the human cytokine genes are regulated by ARE-mediated decay (AMD) [45]. The action of AREs may be considered a mechanism that limits, or buffers, gene expression after production of the mRNA as a response to cellular alterations and extracellular signals. There is relatively little sequence similarity among AREs, but they generally contain AUUUA pentamers and/or U-rich sequences [46]. Even though AREs are primarily characterized by their ability to promote rapid deadenylation of mRNA transcripts, some ARE-containing mRNAs, such as TNFα, are also translationally regulated [47]. The far majority of the key cytokine players in the psoriasis pathogenesis contain AUUUA-pentamers localized in the 3' UTR of the mRNA transcript. An overview of the numbers of potential AREs in cytokine mRNAs involved in the pathogenesis of psoriasis is given in Figure 1; numbers range from a total of 12 AUUUA-pentamers in the 3'UTR of the shared p40 subunit (IL-12B) of IL-12 and IL-23 to one element in IL-19 and TGF-β. Only one of the cytokine mRNAs included in this analysis, encoding IL-21, does not contain the AUUUA sequence. The composition of the 3'UTRs and location of computer-predicted ARE sequences (AUUUA pentamers) is schematically presented for a selected subset of psoriasis-related cytokine mRNAs in Figure 2.

The instability of ARE-containing mRNAs is regulated by ARE-binding proteins (AUBPs) that belong to different protein families and among which some promote destabilization whereas others stabilize mRNAs. These proteins modulate different aspects of mRNA metabolism including deadenylation, exosome binding, or decapping and do not seem to execute the RNA degradation themselves. Instead AUBPs may facilitate mRNA recognition by ribonucleases, including 3'-5' exoribonucleases in the multicomponent exosome complex [48] or 5'-3' exoribonucleases in a more vaguely described non-exosomal degradation pathway [49].

Tristetraprolin (TTP), a member of the TIS11 protein family, is a prominent AUBP that induces mRNA destabilization and has been found to associate with a panel of RNA degradation enzymes. TTP binds the ARE motif (with preference for an RNA segment with the sequence UUAUUUAUU) by means of two tandemly repeated zinc finger domains [50] and was found first to limit production of TNFα in macrophages by inducing destabilization of the mRNA [51]. TTP promotes deadenylation which is required for TTP-directed posttranscriptional regulation [52], and the protein is regulated by phosphorylation through the p38 MAPK pathway (described later). It was recently shown that TTP, among other AUBPs, acts to deliver ARE-containing mRNAs to processing bodies which are cytoplasmic assemblies of mRNAs and components of the RNA decay and silencing machinery [53]. TNFα mRNA is the most studied target of TTP, but a series of other cytokines, including IL-6, IL-10, IL-12, and granulocyte-macrophage colony stimulating factor (GM-CSF) are potential targets with a role in psoriasis pathogenesis. This notion is supported by the wide occurrence of AUUUA-pentamers in cytokine transcripts (Figure 1 and 2) but remains to be studied in further detail. In addition, ARE-containing targets of other TIS11 family members, like BRF-1 [54], include TNFα, IL-3, and GM-CSF. The importance of TTP in regulating cytokine levels is also illustrated by TTP knock-out mice which develop a complex inflammatory phenotype and display inflammatory arthritis, dermatitis, conjunctivitis and myeloid hyperplasia caused by increased cytokine levels, especially of TNFα [55].

Other AUBPs compete with TTP and BRF-1 for binding to ARE sequences. T-cell intracellular antigen-1 (TIA-1) acts to destabilize TNFα mRNA, whereas Hu protein R (HuR) increases the half-life of ARE-containing mRNAs [56]. Other AUBPs like AUF-1, KSRP, and NF90 also affect the stability of ARE containing mRNAs through association with degradation enzymes or components of the exosome [56-61].

#### Regulation of ARE-mediated mRNA decay by p38 MAPK signaling

The mitogen activated protein kinase (MAPK) signaling cascades are major pathways linking extracellular signals to the intracellular machinery to regulate a plethora of cellular processes such as embryogenesis and cell death, stress, differentiation, and proliferation [62]. The cascades contain at least three protein kinases in series culminating in the activation of a MAP kinase. MAP kinases are evolutionary conserved proteins and can be divided into three subfamilies: extracellular signal-regulated kinases (ERKs), c-Jun N-terminal kinases (JNKs), and p38 kinases [63]. These kinases share 60-70% similarity and are activated in response to different stimuli.

The p38 MAPK pathway has long been known to be implicated in the regulation of the biosynthesis of some cytokines [64]. There are four different p38 splice variants (α, β, γ, and δ) with p38α being the best characterized and probably the most physiologically relevant in coordination and regulation of cellular responsiveness to stress [65]. p38 activation can occur by a variety of extracellular stimuli, including proinflammatory cytokines such as IL-1 and TNFα, growth factors, osmolarity changes, UV light, and chemical agents that promote stress responses [66].

Downstream targets of p38α are multiple and include cytosolic proteins, transcription factors, and several kinases that elongate the cascade [67], but one target has been identified that seems to be of particular importance in regards to the involvement of p38α in cytokine regulation, namely the MAPK-activated protein kinase 2 (MK2). MK2 is activated upon phosphorylation by p38α [68] and the first evidence of MK2 being a key enzyme in stress signaling came with the discovery that MK2-deficient mice exhibited increased resistance to lipopolysaccharide (LPS)-induced endotoxic shock [69]. Endotoxic shock is mediated by proinflammatory cytokines and LPS-challenged MK2-deficient mice had more than 90% reduced TNFα serum levels compared to wild type mice. Furthermore, LPS-stimulated cultured spleen cells from these knock-out mice showed a marked reduction in levels of TNFα, IFNγ IL-1β, IL-6, and IL-10.

Since then, numerous studies have been made in elucidating the mechanisms of MK2 involvement in cytokine regulation upon stress stimulation [50,70,71]. p38α is reported to be involved in transcriptional control of cytokines through several mechanisms [72,73], but there is not much evidence of MK2 being directly involved in transcriptional control of cytokines. However, one study shows that MK2 can inhibit heat shock transcription factor 1 (HSF1) by phosphorylation [74]. HSF1 normally represses cytokine promoters [75], and this function is relieved by MK2 activation, thus promoting transcription of cytokine genes.

**Figure 3:**
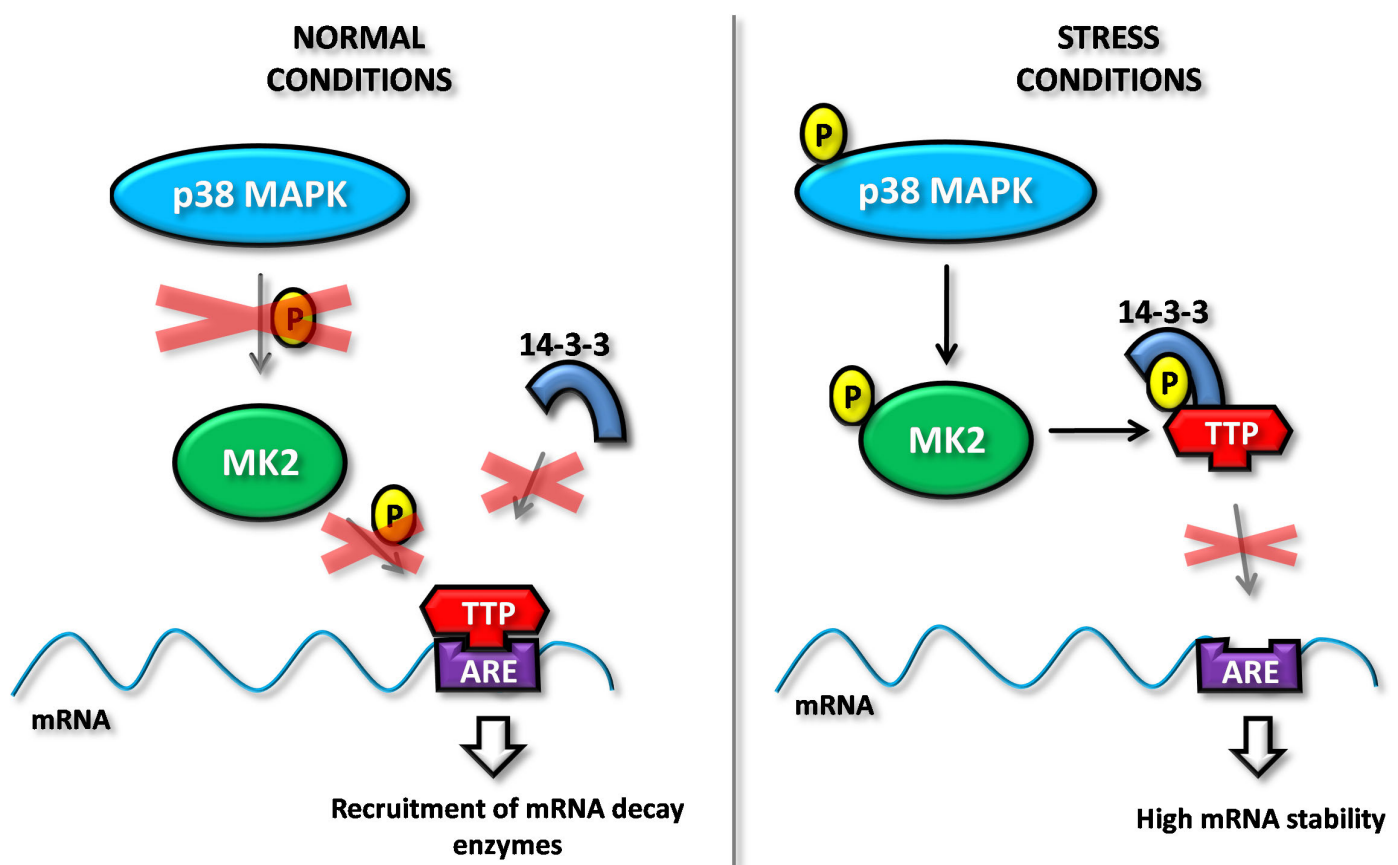
**Overview of the p38-MK2-TTP pathway**. Upon normal cellular conditions the p38 MAPK pathway is not activated, which leaves TTP able to bind the ARE in the 3'UTR of ARE-containing mRNAs and recruit mRNA decay enzymes. Upon a stress stimulus, the p38 MAPK pathway is activated, initiating a phosphorylation cascade which result in the phosphorylation of TTP and a consequent 14-3-3-mediated sequestration of TTP from the ARE.

In contrast, there is much evidence showing that MK2 is involved in post-transcriptional regulation of cytokines including TNFα. The MK2 involvement in ARE-mediated post-transcriptional regulation occurs via TTP, which is, under normal cellular conditions, unphosphorylated and promote rapid ARE-mediated mRNA decay through recruitment of the exosome (Figure 3, left). TTP can be phosphorylated by MK2 at multiple sites [76,77]. Upon p38α activation by stress stimuli, the stress signal is transmitted through a p38α-MK2-TTP phosphorylation cascade (Figure 3, right). Phosphorylation of TTP allows it to interact with 14-3-3 proteins, which are chaperones that stabilize specific conformations of a variety of proteins [78]. The interaction of TTP with 14-3-3 interferes with TTP-mediated recruitment of the exosome and thereby stabilizes ARE-containing mRNAs [79]. The exact mechanism of this has not yet been elucidated. While some studies indicate that TTP exerts its function in cytoplasmic foci called stress granules that arise in response to some types of cellular stress, other studies show that TTP also exerts its destabilizing function in the cytoplasm of non-stressed cells. The main question still exists, if the RNA stabilizing effect of TTP:14-3-3 complex formation is due to physical sequestering of TTP from the RNA or if it is caused by a direct inhibition of exosome recruitment.

Only a limited number of studies have made a direct link between the p38 MAPK pathway and psoriasis pathogenesis. One of the first studies addressing this aspect reported that up-regulated integrins in the suprabasal epidermal layer of psoriatic skin activate various MAPK pathways which in turn lead to unscheduled production of cytokines and growth factors [80]. The link between up-regulated integrins and psoriasis pathogenesis had at that time point already been established and used in a transgenic mouse model of psoriasis in which suprabasal epidermal expression of integrin subunits α2, α5, and β3 led to a psoriatic-like phenotype, including sporadic epidermal hyper-proliferation and a lymphocytic infiltrate [81]. Furthermore, the kinase activity of p38α has been shown to be elevated in psoriatic lesions with a concomitant decrease to normal levels upon resolution of the disease [82].

Later it was shown that also the levels of activated MK2 were increased in psoriatic lesional skin compared to non-lesional skin as measured by the degree of phosphorylated MK2 [83]. Interestingly, the same study reported that increased TNFα protein levels in lesional psoriatic skin were associated with normal TNFα mRNA levels. Down-regulating MK2 in stress-stimulated primary keratinocytes by administering MK2-targeting siRNAs led to significantly reduced levels of TNFα, IL-6 and IL-8 compared to controls. This indicates that increased activation of MK2 may be responsible for the elevated levels of TNFα, IL-6 and IL-8 in psoriatic skin through post-transcriptional regulation and likely through reduction of TTP association with ARE sequences present in these cytokine-encoding mRNAs (TNFα, IL-6 and IL-8 harbor 9, 6, and 9 AUUUA-pentamers, respectively; see Figure 2). Such regulatory mechanisms may apply to the expression of a variety of psoriasis-related cytokines, as suggested by the wealth of potential AREs found in cytokine-encoding transcripts.

The implication of p38α in cytokine regulation has, for more than a decade, been of great interest in regards to development of anti-inflammatory therapeutics. In addition to psoriasis, p38α has been shown to be involved in several other inflammatory condition such as rheumatoid arthritis, Crohn's disease, and asthma [84]. Several small-molecule inhibitors of p38 have been developed which are fairly specific for p38α and p38β [85]. Several of these compounds have proven efficacious in preclinical studies and at least 22 of these have entered clinical studies, however with discouraging results [84]. Some trials were discontinued due to lack of *in vivo *inhibitory potency and inefficient oral bio-availability, but most studies were terminated due to toxicities and significant off-target effects reflecting the multiplicity of pathways and feedback loops in the p38α cascade. Some trials are still ongoing and have completed phase I and II, but there is still quite a lot of skepticism about the clinical value of p38 inhibitors.

The problems regarding p38 inhibition might be circumvented by the use of MK2 inhibitors that can dissect the p38 downstream pathways and interfere only with the inflammatory part without affecting additional cellular functions. Small molecule inhibitors and a peptide inhibitor of MK2 have already been developed showing *in vitro *and *in vivo *preclinical efficacy in inhibiting MK2 involvement in cytokine regulation [86-89]. This lends support to the notion that MK2 inhibition could provide new avenues for anti-inflammatory therapies.

### Fine-tuning gene expression by small RNAs - possible roles of miRNAs in psoriasis

**Figure 4:**
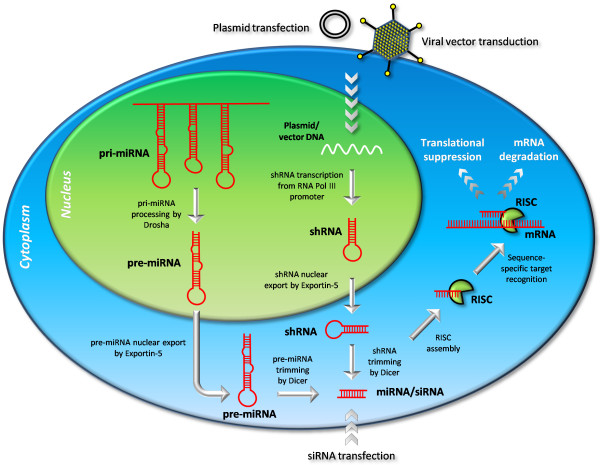
**Overview of RNA interference pathways**. The RNA interference pathway is active in endogenous regulation of gene expression, in which pri-miRNAs are transcribed from the genome and processed in several steps ultimately leading to a mature miRNA which is loaded into the effector protein complex called RISC from where the guide strand of the miRNA guides RISC to mRNAs by sequence-specific target recognition. Depending on the degree of sequence similarity, RISC facilitates either translational suppression or mRNA degradation. The pathway can be exploited for sequence-specific down-regulation of a gene, by transfection of synthetic siRNAs or intracellular expression of shRNAs which are both efficiently processed by the RNAi machinery and enter the RNAi pathway.

An extensive and important group of non-coding RNAs (ncRNAs) appeared with the discovery of miRNAs. More than 700 different miRNAs have been identified in humans and the pathway by which miRNAs exert sequence-specific gene silencing has been elucidated, though numerous questions still remain unanswered. The miRNA pathway was found to converge with the RNA interference (RNAi) pathway [90] by which double-stranded RNA with sequence homology to the mRNA of a particular gene induce post-transcriptional silencing of that gene (Figure 4). The RNAi pathway was first suggested to play a crucial role in a very efficient antiviral mechanism against double-stranded RNA viruses but now, with the discovery of miRNAs, also represent a key path for tuning endogenous gene expression by post-transcriptional regulation.

miRNAs are transcribed mainly from non-coding regions by RNA polymerase II as long primary poly- or monocistronic transcripts called pri-miRNA that are 5' capped and polyadenylated. They can be up to several kilobases long and can contain several distinct miRNAs in short imperfect hairpins. In animals, these transcripts are processed in the nucleus by the endoribonuclease Drosha and the dsRNA-binding protein DGCR8 (a.k.a. Pasha) [91,92]. Drosha cleaves the pri-miRNA in double-stranded stem-loop regions, eleven nucleotides from a hairpin junction, leaving 70-nt hairpins with a 2-nt 3' overhang. These shorter transcripts are called pre-miRNAs and are transported to the cytoplasm by the RanGTP-dependent exportin-5 [93]. In the cytoplasm they are recognized by another endoribonuclease called Dicer which processes the pre-miRNAs by removing the hairpin and trimming the ends, leaving a mature ~21-nt double-stranded miRNA with a 2-nt 3' overhang [94].

The double-stranded miRNA is associated with a class of effector proteins called Argonaute (AGO) proteins [95]. Humans have four of these proteins, AGO1 through 4, of which only AGO2 has been shown to be involved in RNAi and is the only one which exhibits endoribonuclease activity. A potential link between miRNA processing and ARE-directed mRNA degradation is suggested by a possible association of AGO with TTP. The strand with the lower thermodynamic stability at its 5' end remains bound by AGO2 whereas the other strand, termed the passenger strand, is either cleaved by AGO2 or dispelled and degraded in the cytoplasm [96]. The incorporated guide strand and AGO2 associate with other protein factors to form a larger ribonucleic protein (RNP) called the RNA-induced silencing complex (RISC). By means of base-pairing, the guide strand facilitates association of RISC with target mRNA and depending on the degree of sequence similarity between the guide strand and the target mRNA, regulation of gene expression can occur in different ways [97,98]. Perfect or near-perfect sequence similarity promotes mRNA cleavage through the action of AGO2. Less degree of sequence similarity promotes either translational repression by as-yet unknown mechanisms or triggers the transport of the target mRNA to mRNA-processing bodies [99].

In vertebrates, miRNAs mainly target the 3' UTRs of the mRNAs, and the biological basis for this seems to be that active translation of the miRNA target site impairs miRNA inhibitory function [100]. The mRNA recognition occurs predominantly by complete sequence complementarity in 6 nucleotides in the miRNA 5' end (position 2-7) called the 'seed' region, although additional basepairing increases miRNA functionality [98,101-104]. There is rarely full complementarity between the miRNA and the target mRNA, which means that vertebrate miRNA-mediated RNAi happens mainly through translational repression rather than cleavage.

Computational predictions suggest that a single miRNA can target more than 200 mRNAs and, vice versa, that a single mRNA can be under the regulatory control of multiple miRNAs [105]. The substantial involvement of miRNAs in regulating gene expression and the importance of miRNA contribution to essential cellular processes make it no surprise that miRNAs also play a pivotal role in both adaptive and innate immunity, including differentiation of various immune cell subsets and regulation of their immunological functions [106]. The importance of miRNAs in T cell development is illustrated by a study in which miRNA processing was abrogated in mice by a conditional knock-out of Dicer. These mice displayed impaired T cell development and aberrant T_h_-cell differentiation with a preference towards T_h_1 induction and a consequent polarized cytokine production [107]. By computational analysis based on three predictive algorithms, Asirvatham and co-workers searched for miRNA target sites among 613 genes involved in regulating immunity [45]. Interestingly, only 10 out of 34 cytokine-encoding genes included in the analysis were predicted to harbor miRNA target sites, whereas mRNAs encoding AUBPs, crucial effectors of AMD, are potentially heavily targeted by a larger subset of miRNAs [45]. As described later, posttranscriptional regulation by AUBPs and miRNAs may be tightly and directly linked in regulatory pathways in which noncanonical miRNA binding to the mRNA contribute to ARE-directed mRNA degradation.

If not direct regulators of cytokine processing, emerging evidence suggest that miRNAs play a role in regulating the network of cytokine signaling. A wealth of studies link miR-155 to numerous pathways in the immune system, strongly implying that miR-155 is a central regulator of immune function. miR-155-deficient dendritic cells showed impaired ability to activate T-cells [108]. miR-155 has been shown to target suppressor of cytokine signaling 1 (SOCS-1) [109] which negatively regulates the antigen presenting ability of dendritic cells [110]. Higher levels of SOCS-1 in the absence of miR-155 could therefore account for the impaired dendritic cell function. The reverse situation, in which SOCS-1 levels are reduced due to miR-155 up-regulation, could be responsible for uncontrolled dendritic cell function and in fact, in macrophages and dendritic cells, miR-155 has been shown to be up-regulated in response to stress stimulation by LPS [111-113] and several studies report miR-155 up-regulation in patients with rheumatoid arthritis [114,115] - an inflammatory disorder that has an aberrant immune response profile similar to psoriasis.

*In vivo *inhibition of miR-155 by intravenously injected antagomirs, based on locked nucleic acid (LNA), in LPS-treated mice showed a consequent down-regulation of granulocyte-macrophage colony stimulating factor (GM-CSF) mRNA in splenocytes through a yet unknown mechanism [116]. GM-CSF is involved in inflammation and rheumatoid arthritis patients have elevated levels of GM-CSF which correlate with disease severity [117]. Conventional rheumatoid arthritis therapies that antagonize GM-CSF may lead to an abnormally low neutrophil blood count. Hence, the authors suggest that partial antagonism of endogenous GM-CSF by miR-155 inhibition might prove to be a novel therapeutic for the treatment of rheumatoid arthritis and other chronic inflammatory diseases. It still remains unanswered if miR-155 plays a role in psoriasis, but to judge from its central role in general immune function and in rheumatoid arthritis, there is reason to believe that miR-155 might also be implicated in psoriasis.

Like miR-155, miR-146 (miR-146a and miR-146b) has also been found to be up-regulated in dendritic cells in response to LPS stimulation and it has been shown to target two regulators of TNFα signaling, namely IL-1 receptor associated kinase (IRAK1) and TNF receptor-associated factor 6 (TRAF6) [112]. Thus miR-146 seems to be involved in negatively regulating immune-responsive genes which are induced by IRAK1 and TRAF6. To appreciate the implication of IRAK1 and TRAF6 in TNFα regulation, a study showed that RNAi-mediated repression of IRAK1 and TRAF6 in human monocytes resulted in a 85% reduction in TNFα levels [114]. In contrast to miR-146, miR-125b was found down-regulated in macrophages in response to LPS stimulation and it was reported to directly target TNFα mRNA. Hence, down-regulation of miR-125b in response to LPS might be required for proper TNFα production [113].

Careful miRNA profiling of miRNAs in lesional psoriatic skin has been carried out by Sonkoly and co-workers [118]. Interestingly, they found that miR-146 was up-regulated in psoriasis skin, whereas miR-125b was down-regulated [119]. These are possibly secondary effects of the inflammatory signals in psoriatic lesional skin rather than direct contributors to psoriasis pathogenesis. Up-regulated miR-146 in psoriasis is likely to be a negative feedback mechanism in inflammation since it plays an immuno-suppressive role. miR-125b can also be defined as immuno-suppressive since it targets TNFα, but down-regulation of miR-125b in psoriasis is not a protective response, like up-regulation of miR-146, but rather a permissive response. miR-146 and miR-125b also differed in their spatial expression in the various cellular constituents of the skin. miR-125b was mainly expressed in structural cells like fibroblasts, keratinocytes and melanocytes, whereas miR-146 was virtually absent from structural cells, and was instead expressed preferentially by immune cells. Based on their described mechanisms, neither miR-146 nor miR-125b seems to represent therapeutic targets in psoriasis, but supplementing their levels by administration of artificial miRNAs could potentially provide a therapeutic response.

The best described miRNA with connection to psoriasis pathogenesis is miR-203. miR-203 is a skin-specific miRNA expressed predominantly by keratinocytes [119]. miR-203 was found to be up-regulated in psoriasis and computational predictions suggested that miR-203 targets SOCS-3 via an evolutionarily conserved 10-nucleotide sequence that includes the entire seed region and carries perfect complementarity with SOCS-3 mRNA [119]. In fact, miR-203 and SOCS-3 showed complementary expression in psoriatic epidermis: miR-203 was up-regulated and SOCS-3 down-regulated compared to normal skin. SOCS-3 negatively regulates the activation of the transcription factor signal transducer and activator of transcription 3 (STAT-3) [120], which is indeed overly active in psoriasis and implicated in cytokine signaling [121]. Thus, up-regulated miR-203 in psoriasis might have important implications for psoriasis pathogenesis by preventing the up-regulation and negative feedback mechanism of SOCS-3 on cytokine signaling.

However, another study questioned the link between miR-203 and SOCS-3. The authors showed that exogenously expressed miR-203 in murine keratinocytes did not lead to repression of SOCS-3 protein [122]. On the contrary, SOCS-3 levels were slightly up-regulated. However, this study was done in murine keratinocytes and under non-inflammatory conditions, and, thus, it is still possible that miR-203 targets SOCS-3 in human keratinocytes during inflammatory conditions like psoriasis. This seems to be supported by unpublished observations from a luciferase-based reporter assay that validated targeting of the SOCS-3 sequence by miR-203 (described in [123]). In addition to being regulated by miR-203, SOCS-3 carries a destabilizing ARE in its 3' UTR. A study in murine fibroblasts and macrophages showed that stress-induced p38 MAPK activation led to elevated SOCS-3 protein levels. It was also shown that this elevation was mediated by activation of the p38-MK2-TTP axis with a consequent stabilization of SOCS-3 mRNA [124].

### Maintaining the cytokine balance - are miRNAs regulators of ARE-mediated mRNA decay in skin?

Numerous miRNA target sites in cytokine mRNAs are predicted by computational analyses. A small subset of such target sites - predicted with stringent search criteria by two separate algorithms - are listed in Figure 2. Nevertheless, only very few targets have been functionally verified, and further experimental scrutiny is needed to clarify the direct role played by miRNAs during cytokine expression. Pioneering studies, however, suggest that miRNAs are also involved in ARE-mediated regulation of mRNA stability and translation [125]. Such mechanisms are not merely directed by miRNAs targeting ARE motifs by means of the canonical RNAi pathway but may include also miRNA-mediated translational activation and miRNA-assisted TTP binding to its target RNA.

**Figure 5:**
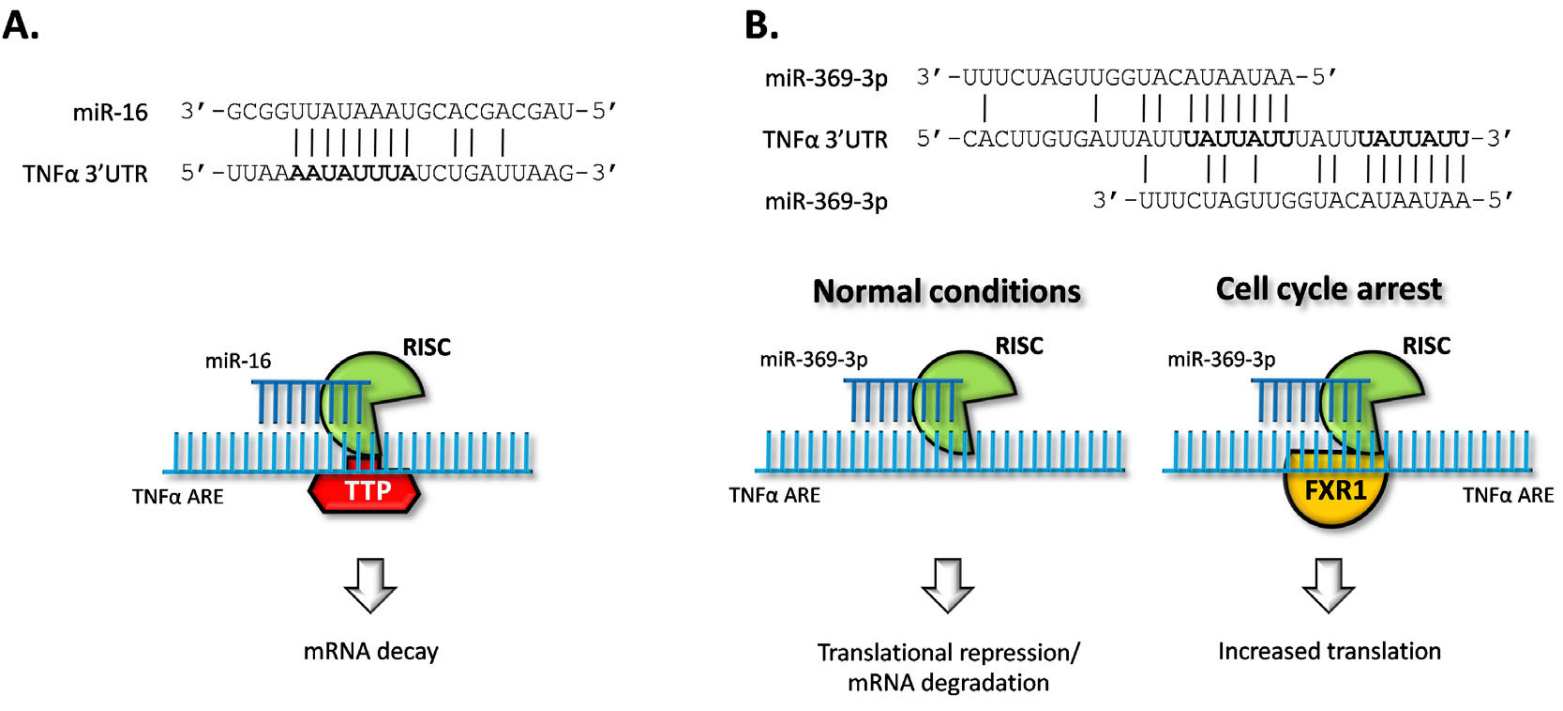
**TNFα regulation by miR-16 and miR-369-3p**. Sequence alignment of part of the TNFα 3'UTR and miR-16 or miR-369-3p. The core, non-seed, recognition sequence is shown in bold. (**A**) TTP is associated with RISC and in a mutually dependent mechanism, TTP and miR-16 promote TNFα mRNA decay through binding to the TNFα ARE. The core non-seed miR-16 recognition motif is shown in bold. (**B**) miR-369-3p promotes canonical translational suppression or mRNA degradation during normal cellular conditions, but upon cell cycle arrest, FXR1 is associated with RISC to increase translational activity. The core miR-369-3p seed match is shown in bold.

miR-369-3p was found by Vasudevan and co-workers to mediate translational activation of TNFα during cell cycle arrest [126]. The TNFα ARE contains two identical 7-nt seed matches to miR-396-3p (Figure 5). The authors showed that miR-369-3p base-pairs with this region and recruits AGO2 and fragile X mental retardation-related protein 1 (FXR1), which are both needed for translational activation. It is important to note that the translational activation was not merely an alleviation of translational repression which corresponds to the translational activity of an ARE-mutated TNFα transcript. AGO2 is the normal effector protein in RISC mediating post-transcriptional repression of gene expression, but its involvement in translational activation actually corresponds to the initial discovery of AGO2 as a translational stimulatory protein [127]. FXR1 is described as a translational regulator that associates with the 60S ribosomal subunit [128], and FXR1-knockout mice have been shown to exhibit cytokine deregulation [129].

Even though the complete mechanism is not fully understood, the finding that two other miRNAs, let-7 and an artificial miRNA, also mediated translational activation during cell cycle arrest suggests that translational regulation by miRNAs oscillates between repression and activation as a function of the cell cycle. More studies will have to be conducted to confirm this, but the described relation between miR-369-3p and TNFα lends support to the notion that miR-369-3p might be involved in aberrant TNFα production and possibly other ARE-containing cytokines. Another study links miR-155 to up-regulated levels of a TNFα 3'UTR-containing reporter [113]. The authors suggest a possible mechanism to be enhanced translation of the transcripts caused by miR-155-mediated suppression of proteins that bind to the TNFα 3'-UTR and repress translation. Whether miRNA-mediated activation of translation is involved in increased TNFα production during inflammation still remains to be explored and it would be an interesting study to investigate if aberrant levels of miR-369-3p are produced during inflammation. Other psoriasis-related cytokines contain the miR-369-3p target sequence (Figure 2), and it is intriguing, therefore, to speculate that miR-369-3p plays a role also in regulating other mRNAs containing the same ARE sequence as TNFα.

Like miR-369-3p, miR-16 also bears a partial sequence match with the TNFα ARE - an 8-bp non-seed match (Figure 5). Several studies confirm that the rapid ARE-mediated decay of TNFα mRNA is mediated by the p38α-MK2-TTP axis (Figure 3) [79], but a recent study showed that miR-16 was also involved in the process and it was shown that TTP and miR-16 are co-dependent in this process [130]. miR-16 inhibition by antagomirs or siRNA-mediated targeting of pre-miR-16 led to a marked increase in TNFα mRNA stability. Furthermore, TTP was identified in RISC bound to the ARE, leading the authors to hypothesize that when bound to the ARE, miR-16-containing RISC assists TTP binding to the ARE which serves to recruit proteins for deadenylation and/or exosomes for mRNA degradation. The authors also investigated the effect on two AREs from other mRNAs and found that miR-16 was only involved in the regulation of one of them. The study demonstrates the involvement of miR-16 in controlling ARE-mediated mRNA decay of some ARE-containing mRNA species and suggests that cooperation of miR-16 and TTP is essential in the recognition of these AREs and in triggering mRNA degradation (Figure 5). The caveats of this study, however, is that it was either performed in drosophila S2 cells or HeLa cells and the effects were only shown on a β-globin reporter gene fused to the AREs. It remains unseen whether production of TNFα in antigen-presenting cells and keratinocytes is influenced by miR-16 and whether this regulatory pathway is affected during skin inflammation. Attempts to modulate miRNA function in keratinocytes in culture should assist in clarifying these issues and development of specific tools for antagonizing miRNA function in xeno-transplanted lesional skin may help define the roles of miRNAs during processing of TNFα and other cytokines. Likewise, it remains a matter of speculation whether the ubiquitously expressed miR-16, through its interaction with AUBPs and noncanonical miR-16 target motifs in multiple cytokines (Figure 2), represents a universal regulator of ARE-directed cytokine regulation.

### Towards the development of small RNA drugs for treatment of inflammatory skin disease

Our knowledge of small RNA-directed gene regulation is constantly growing, and it seems safe to assume that so far we have only seen the very tip of the iceberg with respect to the many potential roles played by small RNA regulators, adding to the enormous complexity of the innate immune system. Inevitably, our understanding of such regulation gives rise to new ideas of exploiting nature's own regulatory pathways in drug development and treatment of inflammatory disease. As we have seen, cytokines are prominent targets for treatment of inflammatory diseases, like rheumatoid arthritis and psoriasis, by systemically administered inhibitors. Do RNA-based drugs represent new means of regulating cytokine signaling during skin inflammation?

In recent years, a plethora of therapeutic possibilities involving the use of double-stranded RNA (dsRNA) as a drug has appeared [131], and several phase I and phase II clinical trials are ongoing (reviewed in [132]). Like pre-miRNAs, exogenous double-stranded RNAs (dsRNAs) are recognized and processed in the cytoplasm by Dicer into ~21-nt double-stranded RNAs termed small interfering RNAs (siRNAs) (Figure 4). Elbashir and co-workers showed first that cellular introduction of a 21-nt duplex RNA was sufficient to mediate sequence-specific gene silencing [133]. This was an important discovery for utilizing the RNAi pathway for sequence-specific gene silencing in studies of gene function and for therapeutic silencing of aberrant gene expression since longer dsRNAs often provoke a severe cytotoxic interferon response [134].

Transfection of synthetic siRNAs has the advantage that the siRNAs do not need to reach the cell nucleus, but it also suffers from the disadvantage that repeated administration is necessary to obtain a persistent effect because the siRNAs are degraded and diluted during cell growth. Sustained production of siRNAs is obtained by integratable or episomally stable vector systems that encode short hairpin RNAs (shRNAs) [135,136]. Like pre-miRNAs, shRNAs are exported from the nucleus by exportin-5 and processed into siRNAs by Dicer. Synthetic siRNAs that are transfected into cells can be taken into the RNAi pathway at this point where they are recognized by AGO2 and loaded into RISC which will mediate gene-specific silencing, occurring mainly by mRNA cleavage due to perfect target sequence similarity. shRNA expression systems represent powerful tools for knocking-down disease genes including gain-of-function single basepair mutant disease alleles. However, care must be taken in the design of these highly discriminating siRNAs and also for the use of siRNAs in general, due to the obvious risk of off-target effects mediated by target complementarity in the seed region of the siRNAs, which reflects natural target regulation by miRNAs [137].

The applicability of locally administered cytokine-directed RNAi therapeutics in skin inflammation seems to depend on at least two key issues. Firstly, cytokine mRNA targets need to be identified and validated in appropriate animal models. Although prime candidates, like TNFα-, IL23-, and IL20-encoding mRNAs, are easily suggested as targets for RNA-based intervention based on their suitability as targets for systemic protein inhibitors, formal evidence for their potential as therapeutic targets during inflammation is required. Secondly, the delivery of RNAi therapeutics to skin remains an extraordinary challenge to any type of cutaneous RNA-directed therapy. A recent review by Geusens provides an excellent view over the possibilities and hopes of RNAi therapy in skin [138]. Here, we will discuss the prospects and major challenges related to cytokine-directed RNAi therapy.

TNFα is produced by skin antigen-presenting cells and by keratinocytes during psoriasis progression and stands out as one of several central cytokines in the pathogenesis of the disease. Recent work by Johansen and co-workers somewhat surprisingly showed that increased levels of TNFα protein in lesional psoriatic skin was not caused by an increase in the levels of TNFα-encoding mRNA but rather by increased translational activity triggered by higher levels of activated MK2 [83]. This suggests that TNFα mRNA would not be perfectly suited as a target for RNAi. However, given the roles played by TNFα we hypothesized in recent work that targeting of TNFα mRNA by local RNAi in the skin would serve as a potential strategy towards treatment of psoriasis [139].

To test this therapeutic approach, we identified one shRNA variant, among a panel of DNA-encoded TNFα-targeting shRNAs, that consistently induced down-regulated expression of TNFα in cultured cells. We and others have shown that transgenic genetic cargo is efficiently delivered to cells of both the epidermis and dermis by intradermally injected lentiviral vectors [139-141]. VSV-G-pseudotyped lentiviral vectors were employed, therefore, to deliver the shRNA expression cassette to lesional psoriatic skin translanted onto SCID mice. By intradermal injection of a single dose of lentiviral vectors encoding TNFα-targeted shRNAs, we measured a reduction in the levels of TNF-α mRNA in transplanted skin and observed both clinical and histological improvements of the psoriasis phenotype. The severity of the psoriasis phenotype, as determined by evaluating the clinical scores of transplanted lesional skin, was improved by the treatment to an extent that was comparable to that observed in mice receiving systemically administered cyclosporin A five times weekly for the duration of the experiment (3 weeks). In addition, anti-TNFα shRNA treatment reduced the epidermal thickness in the skin xenografts. These findings together suggest that TNFα mRNA is in fact a valid target for therapeutic intervention by small RNAs. The data reveal the potential of RNAi therapeutics in treatment of inflammatory skin disease and perhaps most importantly create a new platform for performing systematic screening and evaluation of therapeutic mRNA targets among the many cytokine and cytokine-regulating players involved in psoriasis pathogenesis. Hence, by combining gene-specific targeting with lentiviral delivery it should be possible to target any predetermined gene in transplanted psoriasis skin and evaluate potential phenotypic improvements. On a longer term, such studies are likely to benefit also from the development of vectors that allow simultaneous expression of different shRNAs allowing estimations of the possible beneficial outcome of combinatorial treatments targeting several prime players in disease development.

Despite its efficiency, intradermal administration of lentiviral vectors is currently more an experimental tool than a therapeutic modality in relation to inflammatory skin diseases. Safety considerations, production costs, and the fact that intradermal injection can be applied to only small areas of skin are among the weighty reasons that such treatment is not currently relevant for psoriasis patients. Ironically, the potency of injected lentiviral vectors illustrates some of the major challenges of cutaneous RNAi therapy, namely the delivery of RNA effectors and the ability of these to cross the major barriers of the skin.

As an easily accessible organ, skin stands out an attractive target for RNAi-based therapeutics. However, the skin possesses strong barrier properties that impede easy penetration of topically administered drugs (thoroughly reviewed in [142,143]) and remain a challenge for development of efficaciously delivered RNA drugs. Human skin is composed of a dermis and an epidermis. Cells in the basal layer (stratum basale) of the epidermis at the dermis-epidermis interface proliferate and start to differentiate upon initializing their migration towards the surface of the skin. During migration, the differentiating keratinocytes constitute the stratum spinosum and stratum granulosom. Terminal differentiation occurs at the stratum granulosom, and the migrating keratinocytes are transformed into flat anucleated dead cells, corneocytes, which constitute the densely packed stratum corneum, the outer protective layer of the skin. Cells in this layer are stacked and glued together by lipids embedding the corneocytes. Under normal circumstances, loss of cells from the stratum corneum is balanced by cell proliferation in the basal layer of the epidermis. Underlying the epidermis is the dermis which is penetrated by blood and lymphatic vessels and also embeds the hair follicles and sweat glands.

The tightly packed stratum corneum and narrow intercellular compartments of the skin are the main challenges for drug delivery to skin. Evidently, intradermally injected lentiviral vectors are capable of overcoming both barriers, as cells of both dermis and epidermis are efficiently transduced after a single injection [139]. Similarly, uptake of both plasmid DNA [144,145] and *in vitro *synthesized siRNAs [146,147] by keratinocytes after intradermal injection has been reported and may certainly have some clinical applications. Recent findings have documented targeting of chimeric luciferase reporter RNA molecules by synthetic siRNAs injected into the paws of mice [146,148]. Such data led to the proposal of a clinical trial using siRNAs directed against keratin genes in patients with the rare dominant-negative keratin disorder pachyonychia congenita [149]. The safety and efficacy of a siRNA-based therapeutic for the treatment of pachyonychia congenita was recently evaluated in the first-in-human siRNA clinical trial of an inherited skin disorder [150]. Intradermal injection of TD101, a siRNA that potently targets keratin 6a N171K mutant mRNA, was carried out in a single patient. Adverse effects were not reported, and the RNAi therapy induced regression of the plantar callus which is one of the disabling features of the disease, demonstrating a potential of such treatment that may warrant further clinical investigations.

Despite the promises of intradermally injected siRNA therapeutics or shRNA-expressing viral vectors, a topical RNAi therapy remains the most attractive modality for treatment of inflammatory skin disease. However, a topically applied RNA drug is confronted by the skin barrier and may for some applications depend on penetration-enhancing technologies. Topical administration of RNAi therapeutics is generally considered safe and holds some promise at present as a clinically applicable treatment. Routes of skin permeation go through hair follicles and sweat gland pores or through the narrow intercellular space of the stratum corneum and the remainder of the epidermis. The transport of RNA drugs by these routes may be enhanced by microporation techniques that share the common objective of increasing the permeability of the stratum corneum [151]. Such techniques include the use of microneedles coupled to drug transportation [152,153] or exposure of the skin to ultrasound inducing structural changes in the stratum corneum [154]. Electroporation may also disrupt the structure of skin but has so far typically been combined with intradermal injection [155,156].

Physical methods may be accompanied by chemical approaches including the use of chemicals that disrupt the structure of the stratum corneum or lipid-based systems that are able to penetrate, although often with low efficiency, the outer layers of the skin [157]. GeneCream, a lipid-/alcohol-based formulation developed by Kaspar and colleagues (now at TransDerm Inc., California, USA) stands out as a promising siRNA formulation. Takanashi *et al*. recently reported efficient topical delivery of siRNAs in mouse skin using the GeneCream formulation for delivery of siRNAs targeting osteopontin mRNA [158]. Osteopontin enhances the production of IFNγ and IL-12 in macrophages and has been classified, therefore, as a T_h_1 cytokine with a possible function in the pathogenesis of inflammatory diseases. *In situ *hybridization of siRNAs delivered with the GeneCream formulation showed detectable levels of siRNA in both epidermis and dermis shortly after topical drug administration. In addition, osteopontin-directed siRNAs reduced the levels of osteopontin mRNA in the skin and suppressed the disease symptoms in a mouse model of rheumatoid arthritis [158]. In another ground-breaking report, Ritprajak and co-workers delivered CD86-targeting siRNAs emulsified in baby lotion to the skin of a mouse model for allergic skin disease [159]. CD86 is expressed on the surface of antigen-presenting dendritic cells and plays a crucial role in the activation status of dentritic cells and the antigen-specific T-cell response. Knockdown of the CD86 pathway resulted in reduced homing of dentritic cells in the skin and in the lymph nodes, and, as a result, local inflammation was reduced, demonstrating the potential of targeting cutaneous dendritic cells in the RNA-directed treatment of inflammatory disease. Based on these recent examples in mice, cream-emulsified siRNA delivery seems to match the capacities of viral vectors, even without physical penetration-enhancing methodologies, and certainly holds clinical promise as a noninvasive and patient-friendly treatment of psoriasis and other inflammatory skin diseases. It will be important in the near future to further investigate the permeation of cream-emulsified siRNAs in human skin.

## Conclusions

Another layer of complexity has been added to the circuit of cytokine signaling with the discovery of ARE-mediated mRNA decay and translational control by miRNAs interacting with cytokine-encoding mRNAs. Computational analyses prophesy numerous potential mRNA-miRNA interactions, but only few have so far been confirmed by experimentation, and we are only starting to appreciate the RNA regulatory pathways that are in play during inflammation and inflammatory disease. We do not yet have a feeling with the proportions of the involvement of miRNAs in ARE-mediated decay of cytokine mRNAs, and the roles of miRNAs mediated by noncanonical binding to cytokine-encoding mRNAs have been impossible to foresee by computer-based predictions. Microarray data have clearly shown that certain miRNAs are up- or down-regulated in psoriasis skin [119]. These findings strongly indicate that miRNAs play a role in inflammation and psoriasis pathogenesis [123], and the surface has been scratched with respect to potential roles of miR-203, miR146, and miR-125b. Whether dysregulation of miRNAs during inflammation in skin is a result of unbalanced cytokine signaling or a causative reason for disease development is currently unclear and need further experimental scrutiny. In addition, it is not currently known whether ARE-directed regulation of TNFα is triggered by miRNAs in skin, as was shown for miR-16 and miR-369-3p in human cell cultures, and whether such interactions may apply to other cytokines and could be involved in the pathogenesis of psoriasis.

Our understanding of the molecular mechanisms driving an escalated immune response in skin may give rise to alternative therapeutic strategies for treatment of psoriasis. Current therapies for psoriasis include topical agents, phototherapy and systemic therapies including non-biological drugs, like ciclosporin, or biological therapeutics, like recombinant cytokine receptors or antibodies. Does local targeting of cytokine-encoding mRNAs represent a future possible treatment? We have shown that cytokine mRNAs can indeed be targeted in psoriatic skin and that such artificial regulation of gene expression may induce amelioration of the disease in an animal model. This effect was achieved by intradermal injection of viral vectors encoding TNFα-directed shRNAs and demonstrated that knockdown of a single cytokine gene - despite any potential redundancy - may improve the phenotype. One may speculate if such local treatment - as opposed to a systemic approach - will persistently reduce the influx of T cells in the skin. Indeed, skin-homing immune cells will not be exposed to the drug before entering layers of the skin. In any case, the establishment of such drug regimes awaits the development of effective methods for topical delivery of synthetic siRNAs. We have seen the first examples of such formulations and are eager to learn more about the delivery and applicability of cream-emulsified cytokine-directed siRNAs in human skin and for treatment of psoriasis in the xenograft transplantation model. This seems a very promising approach although it may at present suffer from the high costs related to production of siRNAs.

If the future tells us that miRNAs are major players in inflammatory disorders, a new generation of RNA drugs could include small antisense RNAs that antagonize miRNA function. Such antagomirs can be synthetically made [160] or encoded by DNA [161] and may specifically target an overexpressed disease miRNA. As an example of such strategy, targeting of miR-122 in mouse liver by synthetic stabilized RNA antagomirs has been reported [160] and has recently been followed up by the use of miR-10b-directed antagomirs in cancer treatment including anti-metastatic therapy [162]. As our scrutiny of the cytokine circuit driving skin inflammation is intensified, new therapeutic targets will appear. Despite the current limitations in understanding RNA-directed regulation of cytokines and delivering RNA drugs to human skin, further investigation into cytokine-directed RNA therapy is justified by a vast therapeutic potential.

## Competing interests

The authors declare that they have no competing interests.

## Authors' contributions

The manuscript was prepared by ROB and JGM. Both authors read and approved the final manuscript.
